# Are we divorced from the species we study?

**DOI:** 10.12688/f1000research.2-254.v1

**Published:** 2013-11-22

**Authors:** David Roy Smith

**Affiliations:** 1 Department of Biology, University of Western Ontario, London, N6A 5B7, Canada

## Abstract

In the past few years my interactions with the species I study have been restricted to strings of nucleotides spread across an LCD screen. Bioinformatics has provided me with an amazing window into some of the most interesting algae on Earth, but it has also made me feel distanced from my research organisms, and biology as a whole. This opinion article touches upon these feelings and asks whether many of us should reconsider our relationship to the taxa we investigate.

## A long-distance relationship with *Dunaliella*

Recently, I have been infatuated with *Dunaliella salina*—a fast-swimming, unicellular green alga that can flourish in some of the world’s saltiest waters, and which, given its prodigious lipid content, is being hailed as the ultimate biodiesel factory ^1^. My interests lie in *Dunaliella’*s genomes, the ones in its nucleus, mitochondrion, and chloroplast. For months I have been glued to my laptop computer, bioinformatically piecing together *Dunaliella* genes and measuring genetic diversity among *D. salina* strains collected from remote regions of the planet. My preliminary findings are exciting; I’m uncovering unusual features about this alga’s genomes and how they evolved. But there is one small catch: I have never actually seen, grown, or worked with *Dunaliella*, either in the lab or in the field. My interaction with this salt-loving unicell have been restricted to reading research papers and scrutinizing long strings of nucleotides stretched across an LCD screen. All of this has made me question my relationship to the project and to *Dunaliella*, and I have started to consider that I may be married to the data but divorced from the species I’m studying.

In many ways my experience with *Dunaliella* reflects the current scientific research landscape, which is largely built on collaboration, networking, and outsourcing. This landscape has led to extraordinary global research initiatives and achievements, such as the Human Genome Project and ENCODE, but it has also given rise to inordinate bureaucracy, including author lists that fill entire pages of academic papers and email correspondences that can take hours to sort through. Gone are the days of the all-in-one super scientist, of the self-dependent researcher who could single handedly initiate a major project, execute all of the experiments and analyses, and carry them through to the writing, publication, and communication stages. Today’s fast-paced scientific arena is ruled by teams of specialists, by field experts, bench geeks, and lab managers, by statisticians, computer whizzes, and grant connoisseurs, and by CEO scientists and masters of delegation.

My motivation to explore *Dunaliella* genomes began over coffee and conversation with a friend, Pierre Durand, during an evolution conference at the University of California, Santa Barbara. “Sounds like a plan, Pierre”! I said, with a mouthful of cookie, as we shook hands and hashed out the details. Pierre’s collaborators in Chile had some rare strains of *Dunaliella* isolated from a salt pond in northern Chile’s Atacama Desert, which is among the driest places on Earth. The Chilean strains would be shipped to a colleague in South Africa who would isolate their DNA and pass it along to a high-end genome sequencing facility. Much of the bioinformatics would be outsourced, and I would eventually receive the assembled *Dunaliella* DNA data through email. From there, I would contact other *Dunaliella* research teams, including the United States Department of Energy Joint Genome Institute, to glean genomic data from additional *D. salina* geographical isolates, which I could then use to measure genetic diversity and ultimately write a paper.

As far from the “traditional” research model as this may sound, it is becoming the norm. Science has shifted from a do-it-yourself endeavor to a large, complex, and cosmopolitan affair. That I can sit at my kitchen table in Southern Ontario exploring the inner workings of a green alga from one of the most barren environments on Earth shows just how far science has come, and changed. And overall I think these changes are great. I can certainly make meaningful observations and contributions to *Dunaliella* research through bioinformatic analyses—whether they are done at a field station in the Atacama Desert or a coffee shop in Toronto should make no difference. Many astronomers never actually see the stars and planets they investigate and, similarly, certain microbiologists can only ever indirectly observe the tiny organisms they study, and in both of these examples the scientific explorations and interactions are often confined to computer screens.

But a small part of me believes I should have to see, touch, or be near to the thing I am investigating. This may come from a dated and romanticized view of science. As a child I considered biologists to be synonymous with naturalists—Tilley-hatted adventurers, collecting insects, traipsing after birds, and shouting Latin nomenclature at all the exotic plants in their path. Later, when I went into research, I quickly discovered that many biologists, particularly those of the molecular ilk, abhor the outdoors, and an equally large number have never seen the organisms they study in their natural habitat, if they’ve seen them at all. I wonder how many geneticists could name the source of the nucleic acids (i.e., the species and tissue) that Watson, Crick, Franklin, and Wilkins used to decipher the structure of DNA? (The answer is calf thymus).

Not working directly with the species or thing that one is investigating can sometimes lead to a narrow or naïve understanding of that species or thing. The history of science has taught us that great discoveries are often preceded by years of close examination and immersion by a single researcher with his or her study subject. Primatologist Jane Goodall and Nobel laureate and corn geneticist Barbara McClintock immediately come to mind as examples of independent scientists whose devotion and intimate understanding of their study species resulted in major breakthroughs. But in the contemporary research environment, where someone may sequence the genome of a moss one month and a box jellyfish the next, it is hard not to get the sense that certain scientists don’t have the time (or can’t be bothered) to invest years in learning about the taxa whose genes they are so eagerly sequencing.

For me, not having ever worked with *Dunaliella* could mean that I am overlooking important aspects of the genetic data. One of the interesting observations I’ve made from analyzing *D. salina* DNA is that there is a remarkably low level of genetic diversity in the chloroplast genome as compared to the mitochondrial one (unpublished data). If I had a first-hand understanding of *Dunaliella*—its habitat, its lifecycle, its cellular and physiological quirks—I might be able to link this observation to something substantive about the organism. But in a highly competitive and ever changing research environment, it can be difficult to find the resources and time to develop completely new skillsets for a project that might only last a few months. Moreover, it may not be practical to gain these skillsets when the questions being asked can easily be addressed through collaboration with a world expert in the area.

For all we have gained from “big science”, we might have lost touch with some of the goals and aspirations that inspired us go into research in the first place—for me it was to explore and understand the remarkable diversity of microbial life. I should likely consider bringing a little more “life” into my own laboratory, which largely sits empty with the buzz of a few high-powered computers. I have heard that the salt lakes in Utah are jammed packed with *Dunaliella* species, and that when they are in bloom the lakes light up with a deep pink hue from the beta-carotene of the algae. It is probably worth a trip out there to see for myself—that is, as long as those research grant applications come through.

In fact, maybe all us scientists that are feeling distanced from our research organisms should make an effort to directly interact with them; we certainly could benefit from a more holistic understanding of the amazing species we study, even if we can’t examine each and every aspect in great detail.
