# Supplementary material for: Are we divorced from the species we study?
Source: F1000Res. 2013 Nov 22;2:254. [Version 1] doi: 10.12688/f1000research.2-254.v1 (PMC3869477; doi:10.12688/f1000research.2-254.v1)
Supplement: Peer review history

# Peer review: Referee response for version 1

**Published:** 2013-12-04

**Authors:** Robert Beiko

**Affiliations:** 1 Faculty of Computer Science , Dalhousie University, Halifax, NS, Canada

This is an interesting short piece on the disconnect faced by those of us who focus on computational analysis at the expense of experimental biology. The author suggests that experience with an organism can help with the interpretation of the patterns one finds in its genome(s), and that being totally disconnected from the biology of an organism can lead to a certain ennui, even as one gains exciting new insights into an organism's lifestyle via its genes.

The article is an interesting read; however, it suggests to me that one might consider the value of contributions made by researchers with different areas of expertise. Few can claim to run the gamut of experimental biology including culture, the generation and interpretation of computational data and development and validation of new computational approaches, whilst also managing a lab and an academic career, with all that this entails. In an age of abundant sequence data, one can perform synthesis as never before without ever leaving your desk, apart from the occasional attention paid to the necessities of life. If one wishes to perform broad synthetic work, it may be impossible to connect oneself with all organisms under investigation anyway. The best comparative genomic, evolutionary and ecological meta-analyses (for example) can provide new insights into big questions, producing hypotheses that can be tested with new data that need not be collected by the initial investigator. While it may be more satisfying to some to carry out the entire chain of experiment - big analysis - further experimentation on their own, it is often the case that all the necessary skills, time, or facilities are not available to one person or one research group.

It is true that many of the great biologists (broadly defined) of the past that come immediately to mind, such as Clements, Robert Whittaker, Margaret Dayhoff, Mayr, Baas-Becking, Sanger, and so on, did indeed go on expeditions, work at field stations, grow their own organisms, and purify their own proteins. But if I had one recommendation for this paper, it would be to investigate the matter in more depth, to improve the scholarship by examining the historical contribution of pure theoreticians then and now. Even so, this piece is a worthwhile reflection on the life of those of us who try to make sense of the world without necessarily experiencing it firsthand (except on weekends).

I have read this submission. I believe that I have an appropriate level of expertise to confirm that it is of an acceptable scientific standard.

# Peer review: Referee response for version 1

**Published:** 2013-11-27

**Authors:** Iñaki Ruiz-Trillo

**Affiliations:** 1 Department of Genetics, University of Barcelona, Barcelona, Spain

This is an interesting opinion article in which the author describes a common problem in biological research, especially in the genomics/genetics field. This problem is a lack of knowledge of the biology and/or ecology of some of the species that we, as researchers, analyze at a genetic level. The author describes why much of the current research is like this and asks whether this may limit us to a narrow understanding of the genome itself. Typically the research is done by combining different expertise, so that some researches will understand the biology and the ecology of the species, while others will unravel the contents of the genome. 

The manuscript is very well written and will hopefully make a lot of us think twice about the way we perform research nowadays.

I have read this submission. I believe that I have an appropriate level of expertise to confirm that it is of an acceptable scientific standard.
